# Comparison of Broder’s and Bryne’s Grading System for Oral Squamous Cell Carcinoma With Lymph Node Metastases and Prognosis: A Scoping Review

**DOI:** 10.7759/cureus.51713

**Published:** 2024-01-05

**Authors:** Snega Thamilselvan, Deepak Pandiar, Reshma P Krishnan, Karthikeyan Ramalingam, Pinky Pavithran

**Affiliations:** 1 Oral Pathology and Microbiology, Saveetha Dental College and Hospitals, Saveetha Institute of Medical and Technical Sciences, Saveetha University, Chennai, IND

**Keywords:** prognosticator, oral squamous cell carcinoma, nodal metastasis, bryne’s grading system, broder’s grading system

## Abstract

Oral squamous cell carcinoma (OSCC) has the highest mortality rate of any type of head and neck squamous cell carcinoma. For many eons, the clinical TNM (tumor size, nodal metastasis, and distant metastasis) classification and histological grading of malignancies have been used to predict clinical behavior, confusing it with prognosis and overall survival. This review aimed to systematically identify and evaluate the prognostic value of Broder’s and Bryne’s grading system for OSCC. Electronic resources such as PubMed, Cochrane Database of Systematic Reviews, Google Scholar, Scopus, and direct web searches were used to conduct a thorough search. The titles were examined to identify relevant papers, which were then reviewed for inclusion by reading the abstract. To incorporate studies published outside of the electronic database, the bibliography of all recognized papers was scanned. This review examined all research that investigated the prognostic value of Broder’s and Bryne’s grading systems in OSCC. The electronic database search identified 221 articles. After reading full articles, based on the titles and abstracts and after removing duplicates, six articles were screened. Finally, six articles were selected based on their ability to meet the inclusion criteria and answer the research question. All studies analyzed the competence of this histological grading system in predicting the prognosis of OSCC patients. Four studies evaluated lymph node metastasis and two studies analyzed the histological grading of OSCC. While evaluating the histological grade, we recommend the application of Bryne’s (1992) system for grading OSCC. The standardization of a single, effective method would make it easier to compare results from various studies. This grading system yields better interobserver agreement and bears a prognostic value which may help in devising a treatment strategy for better patient outcomes.

## Introduction and background

Oral cancer is the 16th most common cancer worldwide arising from the mucous membrane of the oral cavity [1]. In India, 162,234 new cases of head and neck squamous cell carcinoma have been reported per year along with 92,141 fatalities, with the prevalence comparatively higher in men than in women. Oral cancer accounts for 16.1% and 10.4% of all cancers in men and women, respectively, in India [2]. Of all oral cancers, squamous cell carcinoma constitutes about 90-95% in India [3]. Oral squamous cell carcinoma (OSCC) is often associated with 47% local recurrence and poor survival. Identifying patients at risk for recurrence has always been challenging as the pathogenesis of OSCC is multifactorial and unpredictable. The prognosis of OSCC is not solely dependent on the clinical TNM (tumor size, nodal metastasis, and distant metastasis) classification but also on histological grading [4,5]. Many histopathological grading systems have been identified to assess the prognosis in OSCC patients.

Different grading systems have been formulated and proven but one primary simpler histopathological grading system is necessary to be tailed rather than shuttling between many. The existing histopathological grading systems include various parameters such as tumor grade, tumor budding, depth of invasion, perineural invasion, lymphovascular invasion, lymphocytic host response, and mitotic activity [6]. For many years, the histological grading of tumors has been used to predict the prognosis and overall survival. The various grading systems for grading OSCC are the Broder grading system (1927), Jakobsson grading system (1973), Fisher grading system (1975), Lund grading system (1975), Willen grading system (1975), Crissman grading system (1980), Anneroth’s grading system (1987), Bryne’s grading system (1989, 1992) and World Health Organization (WHO) grading system. The WHO grading system is based on Broder’s grading system which depends on the resemblance of the tumor cells to parent tissue and the amount of keratin formed. Thus, we chose to compare the application of Broder’s and Bryne’s grading systems in this review as most pathologists apply either of the two grading systems.

**Figure 1:**
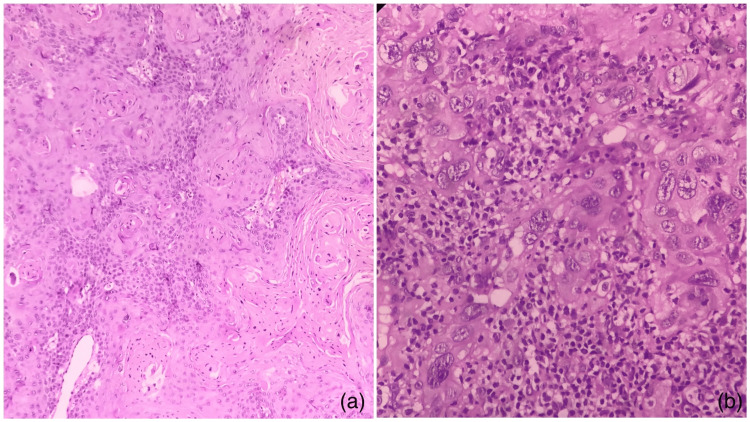
Photomicrographs showing the comparison of cytological and nuclear features in a case of OSCC from our archives showing the superficial part of the tumor (a) which is well-differentiated compared to the invasive tumor front (H&E stain, 10×) and (b) where the tumor cells show marked atypia (H&E stain, 40×). OSCC: oral squamous cell carcinoma; H&E: hematoxylin and eosin

Previous studies have analyzed, compared, and suggested that Broder’s and Bryne’s grading systems are positively correlated with the prognosis in OSCC [5,7,8]. In contrast, other studies have demonstrated contrasting results showing lower prognostic value of Broder’s grading system [4,5,9]. Broder’s grading system considers the resemblance of tumor cells to the oral epithelium in terms of differentiation, whereas, in Bryne’s grading system, the cells are graded at the invasive tumor front (ITF); thus, the same tumor may have different differentiation in the bulk of the lesion and at ITF (Figure 1).

However, it has been shown that tumor prognosis largely depends on the tumor cells at ITF where complex epithelial mesenchymal interaction is noted. Thus, this review aimed to systematically identify and evaluate the prognostic value of Broder’s and Bryne’s grading system for OSCC as histological grading is not only of academic interest but also helps in devising the treatment strategy.

## Review

Methodology

This review was done following the Preferred Reporting Items for Systematic Reviews and Meta-Analyses (PRISMA) guidelines (2020) with a structured question of out of the two most commonly used grading systems of OSCC (Bryne’s and Broder’s), which histological grading system predicts better nodal metastasis and prognosis?

Search Strategy

A systematic search was done on PubMed, Cochrane Database of Systematic Reviews, Google Scholar, Scopus, and direct web search using the search terms “Histological grading,” “Histopathological grading,” “Histopathological grading systems,” “comparison,” “OSCC,” “Squamous cell carcinoma of oral cavity,” and “Oral squamous cell carcinoma” either singly or in combination.

Inclusion and Exclusion Criteria

All full-length articles evaluated both Broder’s and Bryne’s grading system in OSCC as a prognostic evaluator using hematoxylin and eosin (H&E) or immunohistochemistry (IHC)-stained slides. Studies published in languages other than English were excluded. Letters, editorials, reviews, and short communications were also excluded.

Broder’s (1920) Classification

Broder developed a quantitative grading system in 1920. However, classifying OSCC solely on the differentiation or maturation of the tumor cell population has limited utility as a basis for treatment options and prediction of disease outcomes [10]. Accordingly, tumors were graded based on the degree of differentiation and keratinization of tumor cells into four grades. Broder’s grading system is described in Table 1.

**Table 1:** Broder’s quantitative histological grading system based on the differentiation of malignant tumor cells.

Histological grade	Percentage of undifferentiated cells
Well-differentiated (Grade I)	<25% undifferentiated cells
Moderately differentiated (Grade II)	<50% undifferentiated cells
Poorly differentiated (Grade III)	<75% undifferentiated cells
Anaplastic/pleomorphic (Grade IV)	>75% undifferentiated cells

Bryne’s (1989 and 1992) Classification

A multifactorial histological grading system was developed by Bryne et al. in 1989 which includes parameters that identify the invasive capacity of the tumor. Later, in 1992, they modified the grading system by removing one parameter, “number of mitosis” in a high-power field (Table 2). Due to tumor heterogeneity and interobserver disagreement, the validity of the mitotic count as a prognosis marker remains debatable [10]. Each parameter was given an individual scoring, and scoring criteria were also formulated along with it. The scores were summed up and graded.

**Table 2:** Bryne’s histological grading system along with its scoring (1992). The tumor cells are graded at the invasive tumor front. *: not included for grading.

Morphological feature	Score			
	1	2	3	4
Degrees of keratinization	Highly keratinized (>50% of the cells)	Moderately keratinized (20–50% of the cells)	Minimal keratinization (5–20% of the cells)	No keratinization (0–5% of the cells)
Nuclear polymorphism	Little nuclear polymorphism (>75% mature cells)	Moderately abundant nuclear polymorphism (50–75% mature cells)	Abundant nuclear polymorphism (25–50% mature cells)	Extreme nuclear polymorphism (0–25% mature cells)
Number of mitoses per 10 high-power fields*	0–1	2–3	4–5	>5
Pattern of invasion	Pushing, well-delineated, infiltrating borders	Infiltrating, solid cords, bands, and/or strands	Small groups or cords of infiltrating cells (>15)	Marked and widespread cellular dissociation in small groups and/or in single cells (<15)
Lymphoplasmacytic infiltration	Marked	Moderate	Slight	None
Final Score: Grade I (well differentiated): 4–8; Grade II: 9–12 (moderately differentiated); Grade III: 13–16 (poorly differentiated)				

Data Collection and Analysis

A comprehensive search of electronic databases was done and duplicate studies were removed. All articles were initially screened by two researchers (ST and DP) independently based on the aforementioned inclusion and exclusion criteria. Discrepancies between the two researchers were discussed and resolved by consensus. In case of any disagreement, a third author (RPK) was involved to reach a uniform consensus. The abstracts of the studies were evaluated to identify the final studies. The following data were extracted from the studies and analyzed: author(s), year, sample size, parameters analyzed, grading systems, and results and inferences.

Quality Assessment

For assessing the risk of bias in the included six studies, we used the Quality Assessment of Diagnostic Accuracy Studies (QUADAS-2) tool, which rated the studies as “high risk,” “moderate risk,” or “low risk”. The overall risk was assessed based on the patient selection, reference standard, flow, index test, and timing. The Review Manager tool (RevMan version 5.3, The Nordic Cochrane Centre, The Cochrane Collaboration, Copenhagen, Denmark) was used to check the diagnostic accuracy.

Results

Study Selection

**Figure 2:**
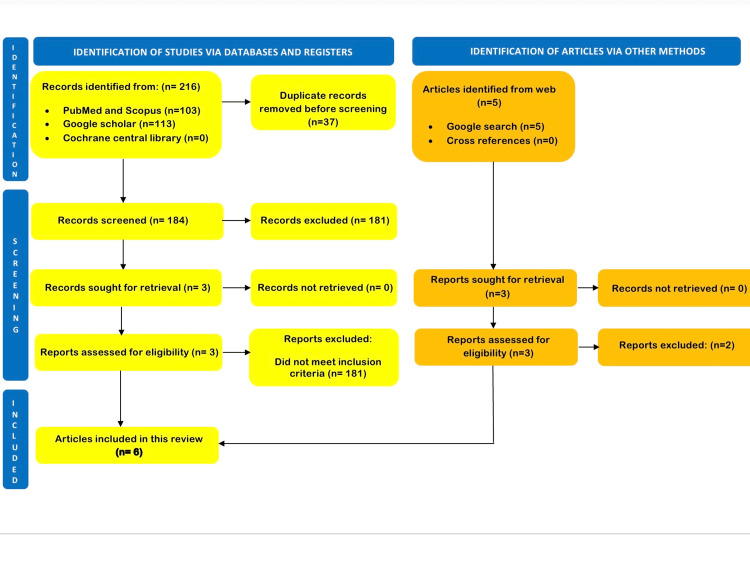
Flowchart of study selection as adapted from the Preferred Reporting Items for Systematic Reviews and Meta-Analyses for the studies included and excluded.

The systematic search from the electronic databases of PubMed and Scopus revealed 103 studies and Google Scholar revealed another 113 studies. After the removal of duplicates and title scans, 184 studies were identified. A total of 181 articles were further eliminated after reading abstract and full texts as these did not meet the inclusion and exclusion criteria. Three full-text articles were identified through cross-reference. The manual Google search yielded another five articles, but after reading the entire text, only three articles were selected. Thus, a total of six studies were finally included [11-16]. Figure 2 depicts the PRISMA flowchart (2020).

Study Characteristics

The detailed study characteristics of the included six studies are summarized in Table 3.

**Table 3:** Demographic and clinical data extracted from the included studies.

Author & year	Sample size	Parameters analyzed	Grading systems	Results	Conclusions
Doshi et al., 2011 [11]	57	Lymph node with metastasis non-metastasis	Broder’s classification (1920). Anneroth’s multifactorial grading system (1987). Bryne’s deep invasive cell grading system (1989)	Poor correlation with the Broder’s grading system and the prognosis or survival. No relationship between Anneroth’s grading system and lymph node metastasis. The invasive behavior of the tumor correlated with lymph node metastasis when analyzed using Bryne’s grading system	Bryne’s grading system for OSCC can be considered valuable in predicting lymph node metastasis
Jamadar et al., 2014 [12]	20	Lymph node metastasis	Broder’s grading system. Jakobsson’s grading system. Bryne’s grading system. Anneroth’s grading system	Lack of correlation between Broder’s grading and prognosis. Positive correlation between Bryne’s grading system and lymph node metastasis	Jakobsson’s and Bryne’s grading system depicted nuclear pleomorphism and pattern of invasion provided the most prognostic characteristics and the keratinization in the ITF added more value for predicting lymph node metastasis
Bedre et al., 2017 [13]	20	Lymph node metastasis	Broder’s grading system. Bryne’s grading system. Anneroth’s grading system	Bryne’s grading system showed a significant relationship with lymph node metastasis when compared with Broder’s and Anneroth’s staging system	Bryne’s system was found to be more informative and valuable in predicting lymph node metastasis
Yazdi et al., 2013 [14]	48	Lymph node metastasis	Bryne’s grading system (1989). Bryne’s grading system (1992). Broder’s grading system. Anneroth’s grading system	Bryne’s grading system with invasive cell grading was found to be highly useful and correlated with lymph node metastasis. Broder’s and Anneroth’s grading system did not correlate with the lymph node metastasis	Bryne’s grading system can be more predictive of nodal metastasis
John et al., 2021 [15]	48	Histopathological grading of OSCC and alterations of ECM regarding the changes in the collagen and elastic fibers	Bryne’s grading systems. Broder’s grading system	The pattern of invasion was considered in Bryne’s grading system. Broder’s grading system was found to be more subjective	Bryne’s invasive front grading system was identified to be more significant for the differentiation of tumor cells
Akinyamoju et al., 2013 [16]	32	Histopathological grading	Broder’s grading system. Bryne’s grading systems	Broder’s classification showed that 31.3% of cases were well differentiated, 50% were MDSCC and 18.7% were PDSCC. Using Bryne’s system, 28% of the cases had a high malignancy score while 72% had a low malignancy score	Either Bryne’s system or Broder’s classification can be used to grade OSCC with similar results being obtained

Lymph Node Metastasis

Our search identified that four studies correlated lymph node metastasis in OSCC patients using Broder’s and Bryne’s grading system [11-14]. The aforementioned studies found that Bryne’s grading system significantly correlated with lymph node metastasis. Bryne’s grading system was considered to be more informative and valuable in predicting lymph node metastasis when compared to Broder’s grading system as poor correlation was noted in all studies between Broder’s grading system and nodal metastases.

Histological Grading

Two studies evaluated only the histological grading of the tumor but did not correlate with prognosis or overall survival [15,16]. One study concluded that both grading systems could be used for identifying the tumor type. Both grading systems exhibited similar results in one study [16]. Another study found Bryne’s grading system to be more significant [15].

Quality Assessment (Risk of Bias and Applicability Concern)

**Figure 3:**
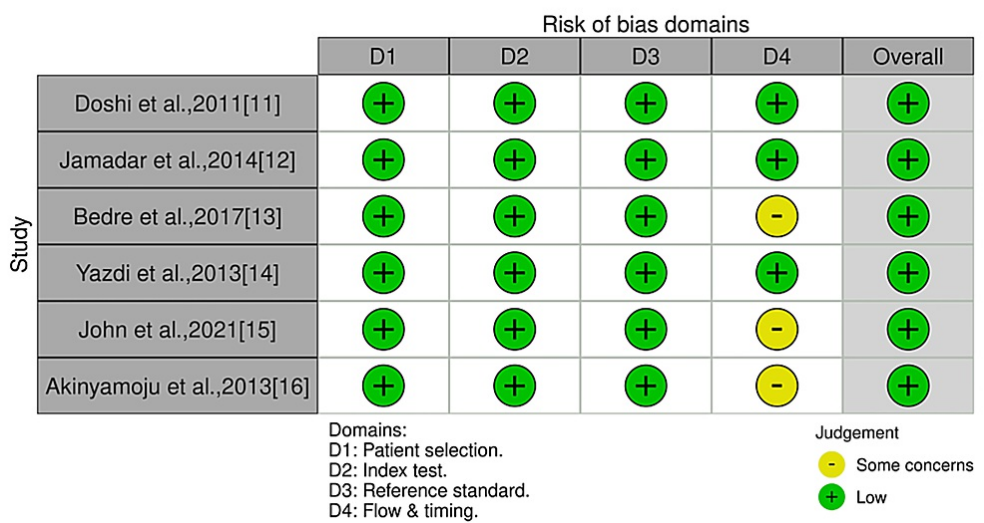
Summary of the risk of bias and applicability concerns for each study (based on the Quality Assessment of Diagnostic Accuracy Studies tool).

**Figure 4:**
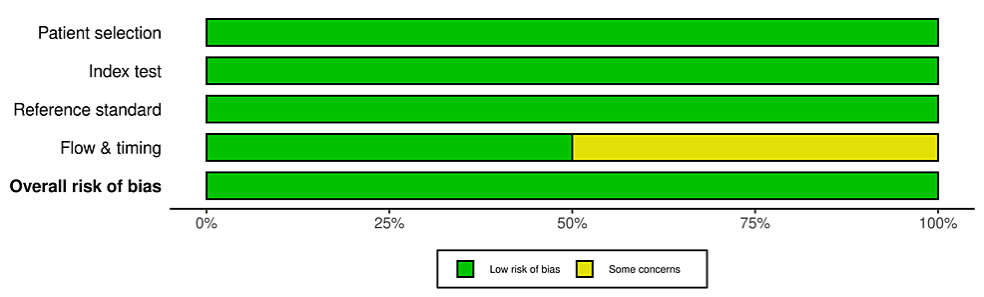
The risk of bias and applicability concerns.

The QUADAS-2 tool showed that all six studies had an overall low risk of bias. Of these six studies, three (50%) demonstrated a low risk of bias in all domains [11,12,14]. The remaining studies had concerns in domain 4, i.e., flow and timing [13,15,16]. Figure 3 and Figure 4 show the quality assessment results of the six studies.

Discussion

OSCC is the most common oral malignant neoplasm with poor patient outcomes [3,17,18]. In recent years, numerous attempts have been made in terms of theragnostics, but unfortunately, the five-year survival of this disease has not improved and severely cripples the patient’s esthetic and overall quality of life [19-21]. Various cancer staging systems have been proposed, but Denox’s TNM staging has been widely accepted and followed for decades after collaboration with the International Union against Cancer and the American Joint Committee on Cancer. This was initially developed for clinical staging evaluation but later the histopathological grading was required for a complete evaluation. The primary goal of staging data is to determine the extent of the tumor and analyze patterns of care and outcomes [10]. Ideally, TNM staging alone is not sufficient in determining the prognosis [22,23]. Experts have been revising the grading systems regularly to find the most effective method of predicting patient outcomes [24].

The histopathological grading is mandatory which includes analysis of cellular and nuclear details of the tumor cells, thereby facilitating the understanding of the biological behavior of the tumor. The first histopathological grading system developed was Broder’s grading system in 1920 [25]. It considered the morphological characteristics of the tumor, but then the tumor-host relationship as well as invasive characteristics were included in the grading system developed by Bryne in 1991 [26]. The primary aim of developing a histopathological grading system is to predict the prognosis in OSCC patients. OSCC has a high proclivity for metastasizing to lymph nodes.

Broder’s grading system has four grades according to the differentiation of tumor cells. The system has been used widely but the limitation was the relationship between the grading and outcome or survival of the patients with OSCC. This is in concordance with several studies. Previously, authors utilized Broder’s grading system and correlated it with lymph node metastasis, but it did not produce any prognostic value concerning prognosis [11,12,14]. This could be because of the difference in the neoplastic nature of the tumor cells [4,5]. Hence, this system of grading may not be adequate for predicting lymph node metastasis. However, it can be used to determine the histological differentiation to predict the tumor type [15,16]. Determining the severity only with the differentiation of the cells and its ability to produce keratin is not sufficient when we consider the prognosis and survival of the patient.

Bryne’s grading system was introduced in 1989 and evaluated the degree of keratinization, nuclear polymorphisms, pattern of invasion, and lymphoplasmacytic infiltration [5]. Later, they modified and excluded the parameter “number of mitosis.” Even without this parameter, the prognostic value remained highly significant. Bryne’s grading system was devised in 1992 and examined the tumor cells at the ITF. The main advantages of this system were (a) it may be used on incisional biopsies, (b) produces high interobserver agreement, and (c) predicts prognosis than any other grading system for OSCC. Treatment failure and poor prognosis in OSCC are primarily caused by recurrence and metastasis. The epithelial-mesenchymal transition (EMT) occurs when cells improve their migratory and invasive abilities. Cells undergoing EMT are more proliferative and lose epithelium-like characteristics to evolve with more mesenchymal cell-like properties. This has been supported by the fact that the tumor cells lose immunoexpression of plakoglobin and e-cadherin and begin to express mesenchymal markers such as fibronectin, vimentin, and smooth muscle actin [27]. EMT also supports the malignant tumor cells to escape anoikis, survive in circulation, and, finally, distant metastases [17].

Bryne’s (1992) grading system was the only system associated with poor disease-specific survival with the abovementioned parameters [24]. All studies that correlated with lymph node metastasis and histological differentiation using Bryne’s grading system demonstrated positive and reliable results over any other grading systems [11,14,16,24]. The authors thought that tumor cell populations in malignancies are heterogeneous, the tumor cells at the deepest portion (ITF) are comparatively more poorly differentiated than the tumor cells in the superficial part of the tumor. Therefore, this grading system employs evaluation of the cells at the ITF [26]. However, it should ideally be evaluated in all invasive fronts or the mean number of invasive fronts should be calculated and evaluated. Thus, the results of this scoping review support the application of Bryne’s histological grading system for OSCC which is a valuable tool for predicting patient outcomes.

## Conclusions

Bryne’s grading system appears to be the best in predicting the outcome of OSCC. It is not only a good prognosticator but also yields higher interobserver agreement. This system involves the examination of tumor cells at the deepest portion of the tumor which are the least differentiated and interact with the host immune system, predicting the overall survival and propensity to show metastasis. When evaluating the histological grade, we recommend that authors apply Bryne’s (1992) classification. The standardization of a single, effective method will make it easier to compare results from various studies.
